# Molecular Characterization of *Acanthamoeba* Isolates from Surface Resting Waters in Northwest Iran

**Published:** 2017

**Authors:** Esmaeil FALLAH, Zahra JAFARPOUR, Mahmoud MAHAMI-OSKOUEI, Ali HAGHIGHI, Maryam NIYYATI, Adel SPOTIN, Aram KHEZRI

**Affiliations:** 1. Immunology Research Center, Tabriz University of Medical Sciences, Tabriz, Iran; 2. Dept. of Parasitology and Mycology, Faculty of Medicine, Tabriz University of Medical Sciences, Tabriz, Iran; 3. Dept. of Parasitology, School of Medicine, Shahid Beheshti University of Medical Sciences, Tehran, Iran

**Keywords:** *Acanthamoeba*, Surface water, PCR, Iran

## Abstract

**Background:**

*Acanthamoeba* is an opportunistic amphizoic protozoan found in different fresh water sources. The aim of this study was to identify and characterize *Acanthamoeba* isolates from surface resting waters, in Northwest Iran.

**Methods:**

Samples were collected from twenty-two different areas, between May and Sep 2014. After filtration, samples were cultivated on non-nutrient agar. The extracted DNAs were amplified and sequenced using partial 18S rRNA in order to genotype and phylogenetic analyses.

**Results:**

Thirty-four (68%) out of 50 collected samples were positive for free-living amoebae based on both culture and morphological characterizations but 28 samples were identified as *Acanthamoeba* spp. by PCR. Sequentially, one isolate was identified as *A. lenticulata*, (T5) (AN: KP940443, identity 99.7%–100%, and divergence 0.3%) whilst other sequenced isolates identified *Acanthamoeba* spp. (AN: KP940444-45) as very similar to *A. rhysodes* and *A. royreba* with identity 100% and divergence 0%.

**Conclusion:**

Surface resting waters in Northwest Iran, were potentially contaminated with pathogenic amphizoic protozoan. Further studies will be required to determine other *Acanthamoeba* species and genotypes in the region.

## Introduction

*Acanthamoeba* species are related to amphizoic and opportunistic protozoa found in the environment. *Acanthamoeba* is found in water and soil, on the skin and other tissues of humans and animals. Recently, due to the advances in diagnostic tools, reported infections of these parasites are extended (1–3). According to their pathogenicity, free-living amoebae are known as reservoirs for bacterial pathogens such as *Legionella pneumophila*, *Mycobacterium avium* and other “amoeba-resistant microorganisms” (4). The size of the *Acanthamoeba* trophozoites is about 25–40μ, but some species have been reported up to 50 μ (5). According to the shape and size of cysts, *Acanthamoeba* species can be classified into three groups (2).

There are two forms of these protozoans in their life cycle including trophozoites and cysts. The cysts have a two-layer wall that protects protozoa against chlorine compounds and antibiotics. This feature produced resistance to temperature ranging from −2 to +45 °C, in the temperature range in which microorganisms such as *Legionella* and amoeba coexist with *Burkholderia* bacteria as they grow and multiply (6–9).

The amoeba cyst is formed in terms of food shortage and other environmental stresses such as temperature and pH changes. Pathogenic species bind to host cells and become virulent (10). Humans become infected through contaminated water, soil or air by trophozoite and the cystic forms of the parasite (6). *Acanthamoeba* species has led to chronic skin infections and central nervous system in patients with impaired immune systems. In addition, it is possible that these patients suffer from corneal ulcers and keratitis. Many cases of keratitis have been reported in people who had a history of swimming in the pool or sea in the region. In addition, some cases of keratitis have been reported in people who drink the water (3, 11). This type of keratitis causes corneal ulcer, severe eye pain (Keratonoritis), photophobia and blindness (12). Therefore, *Acanthamoeba* species can be opportunistic infection in immunocompromised patients. In a report, the five skin infections were observed in patients with AIDS (13).

Due to extensive dispersal of *Acanthamoeba* in the environment, different diseases are caused mostly because of direct contact with water, the examination of resting water contamination seems necessary. A few studies conducted in Iran have focused on tracing *Acanthamoeba* in environmental specimens (14–16) but there is no similar study in this region.

The aim of this study was to identify and characterize *Acanthamoeba* isolates from surface resting waters in Northwest Iran.

## Materials and Methods

### Sample collection

In this cross-sectional study, surface resting waters were collected randomly from public parks and squares in different regions of Tabriz, East Azerbaijan, between May and Sep 2014. Because of uncertainty in the extent of contamination and lack of previous studies, the amount of sampling from each area was 1 L.

### *Acanthamoeba* isolating and culturing

Non-nutrient agar containing *E. coli* was used for culturing and isolation of *Acanthamoeba* as follows: 1.5 g agar was dissolved in 100 ml distilled water, autoclaved and then distributed in plates. Page’s Amoeba Saline was prepared for filtration of the samples. This particular solution contains 0.142 g natrium phosphate, 0.136 g kalium phosphate, 0.12 g natrium chloride, 0.004 g magnesium sulphate, and 0.004 g calcium chloride, which the final volume was adjusted in 1 L by distilled water. The samples were filtered with a vacuum pump and Watman papers (0.42 μ). These papers were put in Page’s Amoeba Saline for 5 min and then centrifuged at 4000 g for 5 min. Sediment at the bottom of the tube was cultivated in non-nutrient agar containing *E. coli* and incubated at 29 °C for 4–7 days. Four days later, the slides were examined using a microscope after being prepared and stained with Giemsa and Trichrome staining (1, 15).

### DNA extraction

DNA extraction was performed by using the commercial Kit (*DynaBio^TM^Takapouzist, Iran*) according to the manufacturer’s instructions.

### Polymerase Chain Reaction (PCR)

For amplification of the partial 18S rRNA gene from *Acanthamoeba* isolates, the specific primers were used describing in previous studies (17): JDP1 forward 5′-GGCCCAGATCGTTTACCGTGAA-3′ and JDP2 reverse 5′-TCTCACAAGCTGCTAGGGGAGTCA-3′. The fragment approximately 500 bp was successfully amplified. PCR reaction was performed in total volume 30 μl including Taq DNA polymerase (1U), Tris-HCL (pH= 9.0)10 mM, each dNTP (dATP, dCTP, dGTP, dTTP) 250 μM, Mgcl2 (1.5mM), KCL (30mM), 10 Pmol from each primer and 5μl (35ng) of template DNA. PCR cycles were set up at 94 °C for 2 min as pre-denaturation step followed by 35 cycles of denaturation 94 °C (35 ses), annealing 56 °C (45 ses), extension 72 °C (1 min) and elongation step at 72 °C (7 min) as final extension. Finally, the amplicons were visualized under UV after electrophoresis on 2% agarose gel and staining with safe stain.

### 18S rRNA sequencing, genotyping and phylogenetic analysis

In this study, in order to genotyping and phylogenetic analysis, 3 isolates of *Acanthamoeba* were sequenced by Genetic Analyzer 3130 ABI. The sequences were compared with GenBank by using Blast (http://blast.ncbi.nlm.nih.gov/Blast.cgi) for determining the species, genotyping and the sequences similarity. Multiple sequence alignment was done by (http://www.ebi.ac.uk/Tools/msa/clustalw2/) and (http://multalin.toulouse.inra.fr/multalin). MEGA 5.05 software and maximum likelihood algorithm with kimura 2-parameter model for phylogenetic analysis were used. The accuracy of phylogenetic tree was evaluated by 1000 bootstrap re-sampling. The sequences pairwise distances (Percent Identity and Divergence) were constructed using the MegAlign program from Laser Gene Bio computing Software Package (DNASTAR, Madison, WI).

## Results

Overall, 34 (68%) out of 50 collected samples were positive for free-living amoebae based on culture and morphology characterization (Table 1).

**Table 1:** The frequency of *Acanthamoeba* isolates from surface resting waters in Tabriz, Northwest Iran

***Sample source***	***No. of sample (%)***	***Culture positive (%)***	***PCR positive (%)***
**Park**	32 (64)	22 (68.75)	20 (62.5)
**Square**	18 (36)	12 (66.67)	8 (44.45)
**Total**	50 (100)	34 (68)	28 (56)

**Fig. 1:**
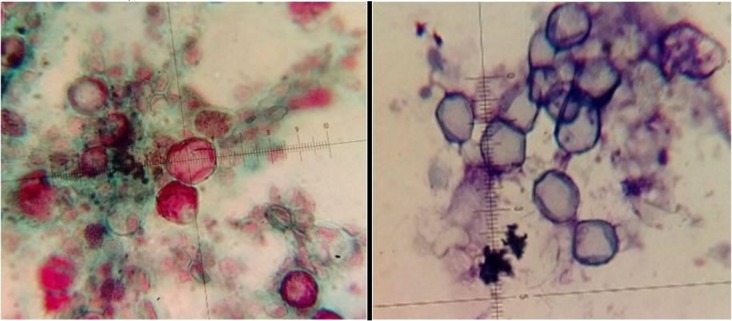
*Acanthamoeba* cysts in primary morphology assessment. Trichrome staining (left), Giemsa staining (right)

**Fig. 2:**
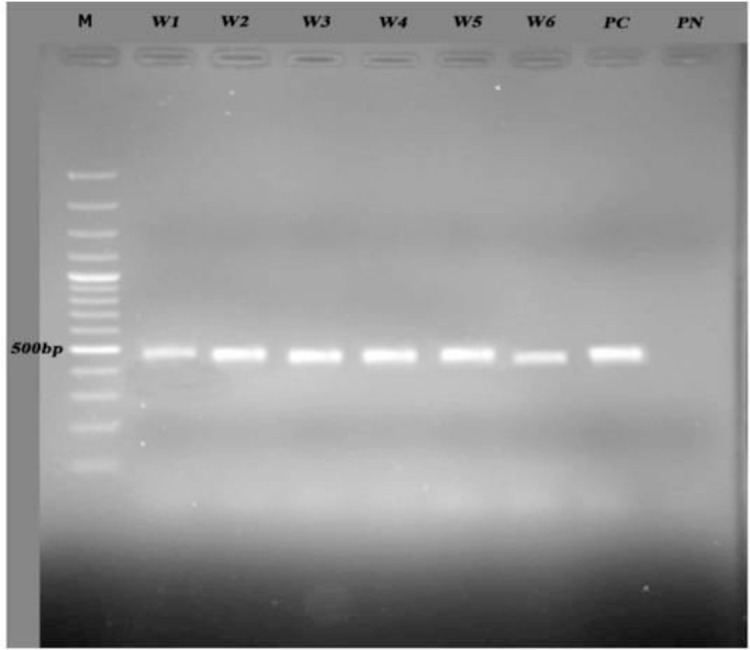
Agarose gel electrophoresis of PCR products. W1–W6: Water samples from different areas. PC: Positive control, PN: Negative control, M: 100 bp DNA size marker

The culture method by non-nutrient agar with bacteria was appropriate for all the samples. Using filter papers and dissolving in Page’s Amoeba Saline were appropriate results in this investigation. Both Trichrome and Giemsa staining methods were used for morphological study (Fig. 1). According to staining, Giemsa with “1 to 5” dilution was suitable and the most appropriate time for staining was 15 min. Polymerase chain reaction was performed using JDP1 and JDP2 primers for small subunit ribosomal DNA and detected approximately 500 bp band on agarose gel for all positive isolates (Fig. 2). Eighty-two percent of all culture positive samples were identified as *Acanthamoeba* spp. by molecular method (Table 1). No specific band was detected in PCR for culture negative samples. Three different isolates after sequencing were submitted and registered in GenBank under the following accession numbers KP940443 (*A. lenticulata,* T5 genotype with identity 99.7%–100% and divergence 0.3%), whilst other sequenced isolates identified *Acanthamoeba* spp. (AN: KP940444-45) as very similar to *A. rhysodes* and *A. royreba* with identity 100% and divergence 0%*.*

**Fig. 3:**
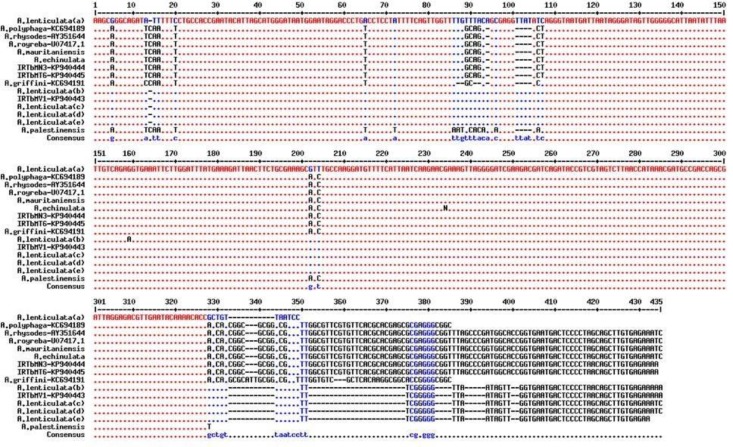
Multiple alignments of the partial 18S rRNA *Acanthamoeba* spp. sequences. *A. lenticulata* (a):KC694187.1, *A. lenticulata* (b):GU573878.1, *A. lenticulata* (c):KC164253.1, *A. lenticulata* (d):U94741.1, *A. lenticulata* (e):KC438381.1, *A. mauritaniensis*:AY351647.1, *A. echinulata*:AY703022.1, *A. palestinensis*: KC694193.1

**Fig. 4:**
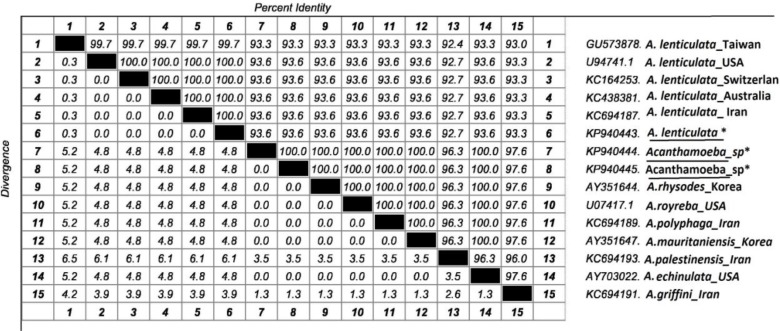
The percent of diversion and identity between the new identified *A. lenticulata*, *Acanthamoeba* spp and selected reference sequences circulating globally from GenBank inferred by partial 18S rRNA. The accession numbers of analyzed sequences in this study characterized by asterisk (*)/underline

**Fig. 5:**
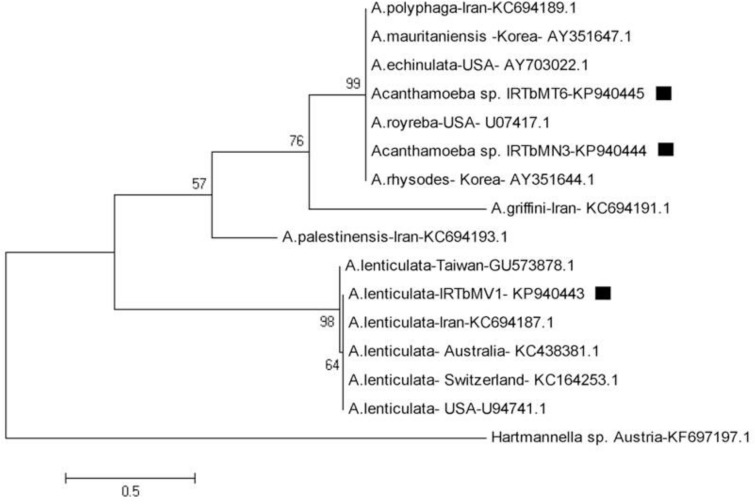
Phylogenetic tree of *Acanthamoeba* 18S rRNA sequences from surface water of Northwest Iran and other previous registered sequences of different areas using maximum likelihood algorithm with kimura 2-parameter model and 1000 bootstrap resampling

Similarities and differences between these sequences and other sequences were previously recorded in GenBank and specified by multiple sequence alignment (Fig. 3, 4). In phylogenic analysis, *A. lenticulata* of this study relied on a branch with similar isolates registered from other countries. The branch including two other isolates showed numerous species such as *A. rhysodes*, *A. royreba* which were very similar to each other. *Hartmannella* sp. (KF697197) was considered as a group branch in drawing the phylogeny tree (Fig. 5).

## Discussion

This study indicated the presence of *Acanthamoeba* spp. in resting waters in Tabriz, Northwest Iran. The findings also showed that the high percentage of resting waters was contaminated and it could be a prominent factor in human infections. In a previous study in Iran, the presence of *Acanthamoeba* spp. was proven in different environmental sources such as water, soil, dust, and cow faeces (14). *Acanthamoeba* spp. has also been isolated from drinking waters of several hospitals in Iran; the prevalence of *Acanthamoeba* in Ahvaz hospitals was reported as 50% (16). These parasites were also isolated from the biofilm in Tehran hospitals where immune deficient patients were hospitalized, and their genotypes mostly belong to T4 (18).

In a similar study on 50 samples of water resources in parks and public squares, of the 22 districts of Tehran, 16 samples (32%) were positively determined by analyzing the sequence and the positive samples belonged to genotype T4 and T5 (19). In another investigation in Thailand, rural natural springs of 13 provinces were contaminated by free-living thermo-stable amoeba, including *Naegleria* and *Acanthamoeba*, which in total, 38.2% of the samples were positive and the water temperature was about 28 - 56 °C (20). After filtering the water samples, filter papers shaking in Page’s Amoeba Saline and then precipitation was transferred to the culture medium (14), which was in line with other studies (21, 22). In Tehran, 37 (46.25%) of 80 samples were contaminated by *Acanthamoeba* (15). A high percentage of *Acanthamoeba* spp. in water, soil, and other environmental samples is a hygienic risk for public health, especially for immune-deficient individuals and contact lens wearers (23). “ *Acanthamoeba* genotype T4 has been isolated from patients with keratitis in Iran” (16). The presence of *Acanthamoeba* spp. in water where human activity is high may cause infection in contact lens wearers (24).

In this study, 34 (68%) out of 50 samples were positive for free-living amoebae based on culture method but 28 samples indicated a specific band for *Acanthamoeba* spp. in PCR. In a similar study in Qazvin, 32 out of 40 samples (80%) were positive for free-living amoebae using the culture method but 14 (43.8%) of culture, positive samples were confirmed by PCR (17). This difference in the results of culture and molecular methods may be due to the presence of free-living amoebae other than *Acanthamoeba* spp. Another study was conducted in Tehran on 16 samples (32%) which were positive (19). The frequency of free-living amoebae with culture in Qazvin was higher than that of Tehran and Tabriz. This difference in frequency may partly relate to the season of sampling, therefore, in Qazvin, it was performed in autumn whereas in Tehran, like the present study, it was in spring and summer seasons. The frequency diversity of isolated *Acanthamoeba* was reported according to the sampling seasons (25, 26). The other factor involved in frequency diversity among Qazvin, Tehran, and Tabriz may be due to the number of samples to be examined; in such a way that in Qazvin, it was two times more than that of Tehran and Tabriz.

Although several genetic markers were used for molecular identification of *Acanthamoeba* isolates, 18S rRNA is known as the most appropriate and useful marker for this purpose (27). T_1_, T_2_, and T_4_ genotypes were related to the causative agent of amoebic encephalitis and keratitis but the dominant genotype in clinical and environmental samples was T_4_ (14, 24, 28). Pathogenic genotypes of *Acanthamoeba* include T_3_, T_4_, and T_5_. In addition, the most frequent genotype isolated in Iran is the T4 (29). However, in the present study, sequence analysis revealed the existence of *A. lenticulata* (T5 genotype) in the water of Northwest Iran. In addition, two other sequenced isolates identified *Acanthamoeba* spp. as very similar to *A. rhysodes* and *A. royreba,* so that there is a difference of only one nucleotide position based on the cyst characteristics. More matches were found with *A. rhysodes*. In a molecular study in Sarein, hot spring of Ardabil Provinces was found that 42.9% of the water was contaminated with *Acanthamoeba*, T4 genotype and Vahlkampfiid amoebae (30).

*A. lenticulata* and *A. rhysodes* can play an important role in human infections, especially people with immune deficiency. Therefore, those similar investigations in other countries were conducted to know the species of *Acanthamoeba* that cause diseases. For example, a case of amoebic infection by *A. lenticulata* (T5 genotype) that a 39-year-old man from Martinique was reported to receive heart transplant (31). In another case, a 28-year-old patient that used hard contact lens with a history of swimming had a corneal abrasion leading to redness; pain and blurred vision. Subsequent tests were taken especially the biopsy of the cornea; it was found keratitisis caused by *A. lenticulata* (32). *A. rhysodes* can also cause eye and CNS infections. Gardner et al. recorded an infection caused by *A. rhysodes* (33). Therefore, according to these explanations; *A. lenticulata* isolated at the first time in this region should be considered as an important potential risk to public health.

## Conclusion

Surface resting waters in Tabriz, Northwest Iran, were contaminated with pathogenic amphizoic protozoan of *A. lenticulata* and *Acanthamoeba* spp. therefore the most effective ways to prevent the human infections seem indispensable. By considering the results of this study and the important role of this protozoan on public health, further studies will be required to determine *Acanthamoeba* species and genotypes in the region.
